# Duhuo Jisheng Decoction suppresses apoptosis and mitochondrial dysfunction in human nucleus pulposus cells by miR-494/SIRT3/mitophagy signal axis

**DOI:** 10.1186/s13018-023-03669-w

**Published:** 2023-03-08

**Authors:** Wei Liu, Xiaolong Zhao, Xuejian Wu

**Affiliations:** 1 Department of Orthopedics, The First Affiliated Hospital of Zhengzhou University, Zhengzhou, 450052 China; 2 Department of Orthopedics, First Hospital of Wuhan, Wuhan, 430022 China; 3 Department of Burn and Repair Reconstruction Surgery, The First Affiliated Hospital of Zhengzhou University, Zhengzhou, 450052 China

**Keywords:** Intervertebral disk degeneration, miR-494, **SIRT**3, Mitophagy, Duhuo Jisheng Decoction

## Abstract

**Background:**

Increasing evidence suggests that mitophagy is responsible for the pathogenesis of intervertebral disk (IVD) degeneration. Previous studies have shown that Duhuo Jisheng Decoction (DHJSD), a classic Fangji of traditional Chinese medicine, can delay IVD degeneration; however, its specific mechanism of action is unknown. In this study, we investigated the mechanism by which DHJSD treatment prevented IVD degeneration in IL-1β-treated human nucleus pulposus (NP) cells in vitro.

**Methods:**

Cell Counting Kit-8 was performed to explore the effects of DHJSD on the viability of NP cells exposed to IL-1β. The mechanism by which DHJSD delays IVD degeneration was explored using luciferase reporter assay, RT-qPCR, western blotting, TUNEL assay, mitophagy detection assay, Mito-SOX, Mitotracker and in situ hybridization.

**Results:**

We observed that DHJSD enhanced the viability of NP cells treated with IL-1β in a concentration-time dependent approach. Moreover, DHJSD lessened IL-1β-induced NP apoptosis and mitochondrial dysfunction and activated mitophagy in NP cells treated with IL-1β. Mitophagy suppressor cyclosporin A reversed the beneficial impacts of DHJSD in NP cells. In addition, the differential expression of miR-494 regulated IL-1β-induced NP apoptosis and mitochondrial dysfunction, and the protective impact of miR-494 on NP cells treated with IL-1β was achieved by mitophagy activation, which was regulated by its target gene, sirtuin 3 (SIRT3). Finally, we observed that DHJSD treatment could effectively delay IL-1β-induced NP apoptosis by affecting the miR-494/SIRT3/mitophagy signal axis.

**Conclusions:**

These results show that the miR-494/SIRT3/mitophagy signaling pathway is responsible for the apoptosis and mitochondrial dysfunction of NP cells and that DHJSD may exert protective effects against IVD degeneration by regulating the miR-494/SIRT3/mitophagy signal axis.

## Introduction

Low back pain (LBP) due to intervertebral disk (IVD) degeneration is very common in modern society, causing a heavy medical and socioeconomic burden [1, 2]. Studies have shown that the nucleus pulposus (NP) is the center of IVD function and the earliest site of degeneration during IVD degeneration and that excessive apoptosis of NP cells (NPCs) leads to a decreased number of cells, which is an important pathological basis of IVD degeneration [3, 4]. However, the specific mechanism leading to the apoptosis of NPCs remains unclear. In mammals, the main function of the mitochondria is energy synthesis and coordination of biological activities that regulate active oxygen production, cell metabolism and apoptosis [5–7]. Studies have shown that the mitochondrial apoptosis pathway is involved in the apoptosis of NPCs [8, 9]. Mitochondrial apoptosis pathways are activated by various factors, resulting in increased permeability of the external membrane of the mitochondria and the release of cytochrome C (Cyt-c) into the cytoplasm, which binds to apoptosis activators and activates caspase-3, leading to a cascade of reactions that result in apoptosis [10]. Reportedly, oxidative stress (OS) caused by IL-1β and other factors may increase the mitochondrial apoptosis pathway of NPCs resulting in IVD degeneration [11–13]. Therefore, inhibition of NPC apoptosis induced by IL-1β can be an important therapeutic target for treating IVD degeneration.

With the intracellular degradation of cytoplasmic macromolecules and dysfunctional organelles, autophagy is crucial for cell homeostasis and survival under stressful conditions. This homeostasis course involves isolation of the cytoplasmic components of the biomembrane autophagosome, fusion of the autophagosome with the lysosome and digestion of the cargo in the lysosome referred to as autophagy flux [14]. A special type of autophagy, mitophagy, selectively degrades damaged mitochondria through the autophagy pathway to maintain the homomorphism of mitochondrial dynamics and reduce mitochondrial dysfunction during OS [15]. Mitophagy dysfunction has been implicated in many degenerative disorders such as IVD degeneration, neurodegenerative disorders and osteoarthritis [16, 17]. The basal level of mitophagy maintains the stability of NPCs. During OS, mitophagy is activated to clear damaged mitochondria and reduce the apoptosis of the mitochondrial pathway of NPCs [18]. Therefore, mitophagy activation can effectively delay the progression of IVD degeneration.

Sirtuin 3 (SIRT3) is located in mitochondria, which is a kind of protein deacetylase. Its activity depends on the auxiliary group nicotinamide adenine dinucleotide, and it is involved in regulating senescence, apoptosis, autophagy and mitophagy. A study by Wang et al. showed that SIRT3 knockdown aggravated apoptosis, senescence and mitochondrial dysfunction, whereas SIRT3 overexpression exerted opposing effects in the NPCs treated with tert-butyl hydroperoxide [19]. A recent study confirmed that SIRT3 is an important protective agent against osteoarthritis by increasing the autophagic flux [20]. Mitophagy mediated by SIRT3 plays an important role in the pathophysiology of IVD degeneration as an important link in clearing mitochondrial reactive oxygen species (ROS) and maintaining mitochondrial stability. The regulation of SIRT3 expression and its mediated mitophagy can play a significant role in delaying IVD degeneration [21]. More and more evidence showed that microRNAs regulate the biological process of NP cells and play an important role in IVD degeneration. We previously confirmed that miR-494 was significantly overexpressed during the progression of IVD degeneration. Downregulating miR-494 can delay the apoptosis of NPCs and the degeneration of the extracellular matrix and promote the repair of intervertebral disks [22]. However, the functional activity of miR-494 in NPCs still needs further investigation.

Several studies have shown that traditional Chinese medicines (TCM) are useful in treating LBP alongside other significant clinical effects [23, 24]. Duhuo Jisheng Decoction (DHJSD), derived from “Bei Ji Qian Jin Yao Fang” of the Tang Dynasty, is composed of 15 Chinese herbs including Radix glycyrrhizae, Panax ginseng, Radix achyranthis bidentatae, Eucommiae ulmoidis cortex, Poria cocos, Cortex cinnamomi, Radix paeoniae alba, Radix rehmanniae, Radix angelicae sinensis, Rhizoma chuanxiong, Herba Asari, Radix saposhnikoviae, Radix gentianae macrophyllae, Ramulus loranthi and Radix angelicae pubescentis, and the main active ingredients are osthol, gentiopicroside, loganic acid and paeoniflorin. The combination of these herbs can eliminate pathogenic factors and play the role of removing rheumatism, relieving arthralgia, tonifying the liver and kidneys and supplementing qi and blood. At present, DHJSD is broadly employed in the clinical treatment of lumbar disk herniation because of its anti-inflammatory and analgesic effects [25, 26]. A meta-analysis showed that modified DHJSD had a more favorable effect on the treatment of lumbar disk herniation than Western medicine, and there were no obvious adverse events [25]. Studies have shown that DHJSD delays the SDF-1-induced inflammatory response by regulating the CXCR4/NF-B signaling axis, thereby delaying IVD degeneration [27]. Our previous study demonstrated that DHJSD delayed the compression-induced degeneration of NP extracellular matrix and NPCs apoptosis by activating autophagy [28]. However, the effect of DHJSD on mitophagy and mitochondrial apoptosis of NPCs requires further investigation.

In the pathological process of IVD degeneration, the NPC produces excessive inflammatory factors, which triggers the subsequent degeneration process, among which IL-1β and TNF-α are the most widely reported inflammatory factors. Our previous research had successfully established the degeneration model of NPCs by using IL-1β [22, 29]. In accordance with the relevant literature, we used IL-1β to make the degeneration model of NPCs in this study. Then, DHJSD was used to intervene in degenerated NPCs, and the effect of DHJSD on IL-1β treated NPCs was detected. We observed that DHJSD could reduce mitochondrial dysfunction and apoptosis of NPCs caused by IL-1β through activating mitophagy. Our study has revealed that **SIRT**3 constitutes the target gene of miR-494, which affects IL-1β-induced NPC apoptosis through **SIRT3-regulated** mitophagy. Finally, we observed that DHJSD suppressed the IL-1β-induced NPC mitochondrial apoptosis by regulating the miR-494/**SIRT**3/mitophagy signaling pathway. Our study highlights the important role of the miR-494/**SIRT**3/mitophagy signal axis in IVD degeneration and explores the anti-apoptotic mechanism of DHJSD on NPCs.

## Materials and methods

### Materials and reagents

DHJSD comprised 6 g each of *Radix glycyrrhizae*, *Panax ginseng*, *Radix achyranthis bidentatae*, *Eucommiae ulmoidis cortex*, *Poria cocos*, *Cortex cinnamomi*, *Radix paeoniae alba*, *Radix rehmanniae*, *Radix angelicae sinensis*, *Rhizoma chuanxiong*, *Herba Asari*, *Radix saposhnikoviae, Radix gentianae macrophyllae*, *Ramulus loranthi* and 9 g of *Radix angelicae pubescentis*. The above-mentioned herbs were provided by the First Hospital of Wuhan. The specific preparation method of DHJSD has been previously reported [28]. It was formulated by the Pharmacy Department of the Wuhan First Hospital to contain 1 g/mL of crude drug. The stock solution was cooled at room temperature and stored at 4 °C prior to usage. In subsequent experiments, we diluted the DHJSD stock solution to ultimate contents of 500, 400, 300, 200 and 100 μg/mL, adopting DMEM/F12 medium with 15% fetal bovine serum (FBS).

### Sample acquisition

We collected NP tissue of 10 patients (5 females and 5 males, aged 11–18 years) undergoing surgery for idiopathic scoliosis in the Wuhan First Hospital from August 2021 to January 2022. All patients were examined by MRI before surgery and graded based on the Pfirrmann grading standard. Of the 10 samples acquired, five were grades I and II, respectively; they were all normal NP samples.

### Isolation and incubation of NPCs

The collected NP tissues were cut into 0.5 × 0.5 × 0.5 mm^3^ tissue pieces and treated with 0.2% type II collagenase for 4 h at 37 °C. The cell suspension was placed in a low-speed centrifuge and centrifuged for 3 min at 155 × g, after which the cells were collected. Then, the NPCs were cultured in a culture flask with DMEM/F12 medium containing 15% FBS and placed in a 5% CO_2_ and 37 °C incubator. The medium was altered every 2 ~ 3 days, and when it reached 80 ~ 90% confluence, we used 0.25% trypsin–EDTA solution for passage. The first generation of NPCs was used for subsequent cell viability testing and intervention experiments.

### NPCs viability assay

The Cell Count Kit-8 method (Dojindo, Japan) was used to determine how DHJSD affects NPC viability. We trypsinized the good growth state of NPCs, inoculated them in a 96-well plate (5 × 10^3^ cells per well) and then incubated them at 37 °C for 24 h. After the completion of the adsorption course, we treat the NPCs with or without IL-1β (10 ng/mL) (Sino Biological Inc., North Wales, PA, USA) and different DHJSD concentrations (500, 400, 300, 200 and 100 μg /mL) for 24 h or various treatment periods (72, 48, 36, 24 and 12 h) at the same concentration (300 μg/mL). The cells were washed at a certain time with PBS, and 100 μL of DMEM containing 10 μL CCK-8 solution was added to all wells and incubated at 37 °C for 2 h. After that, we measured OD at a wavelength of 450 nm using the plate spectrophotometer (Thermo Scientific, USA) to calculate the vitality of NPCs.

### Experimental design and cell treatments

First, we treated the NPCs with DHJSD (300 μg/mL) for 24 h before administrating IL-1β to investigate the effect of DHJSD on NPCs. After that, we pretreated NPCs with DHJSD (300 μg/mL) alone or combined with cyclosporin A (1 μM) for 24 h before IL-1β administration. To explore how miR-494 affected NPCs, we designed a miR-494 inhibitor and mimic and their negative control and synthesized them through GenePharma (Shanghai, China). They were then transfected into NPCs using lipofectamine 2000 before IL-1β administration. To knock down **SIRT**3 expression, scrambled siRNA (siScr) and short interfering (si) RNA targeting **SIRT**3 (si-sirt3) were designed and bought from GenePharma (Shanghai, China). The NPCs were co-transfected by adopting miR-494 suppressor (150 nM) and si-sirt3 (100 nM) for 48 h using lipofectamine 2000. Finally, NPCs were either treated with DHJSD alone or pretransfected with miR-494 mimic (50 nM) or si-sirt3 (100 nM) for 24 h to explore the role of mir-494/**SIRT**3/mitophagy signal axis on DHJSD activity.

### EdU (5-Ethynyl-2′-deoxyuridine) assay

Here, 3*10^4^ cells of NPCs were seeded in 24-well plates. Cells were treated with IL-1β (10 ng/mL) with or without DHJSD (300 μg/mL) for 24 h and then tested following the manufacturer’s instructions (BeyoClick™ EdU Cell Proliferation Kit with Alexa Fluor 594). DAPI was used to stain the cell nucleus before observation. Images were captured under a microscope (Olympus).

### Luciferase reporter assay

The bioinformatics algorithms and related databases were used to search for the potential targets for miR-494. **SIRT**3 was confirmed to have an assumed binding site on miR-494. Wild-type (WT) and mutant (MUT) 3′-UTR fragments containing the assumed miR-494 binding site were amplified and inserted into the pGL3 vector (RiboBio). HEK 293 cells were seeded in a 6-well plate, grown in an incubator at 37 °C for 24 h with 5% CO_2_ and then co-transfected with 100 ng of pGL3 vector harboring MUT 3′-UTR or WT and 40 nM of miR-Scr or miR-494 mimic employing transfection reagent, lipofectamine 2000. The cells were harvested in 48 h to detect luciferase activity through a dual luciferase reporter assay kit (Promega, Madison, Wisconsin, USA).

### Real-time quantitative PCR (RT-qPCR)

The RNA of NPCs was extracted by applying the TRIzol reagent (Invitrogen) based on the manufacturer’s instructions. The RNA content was determined at a wavelength of 260 nm using a spectrometer. Reverse transcription of 1 μg total RNA was used for synthesizing cDNA, and a reaction volume of 10 μL (4.5 μL diluted cDNA, 0.25 μL primers and 5 μL 2 × SYBR Master Mix) was used for PCR amplification. The cycle threshold was recorded. The target gene expression level was normalized to the GAPDH level, and the miR-494 level was normalized to that of U6. The expression of **SIRT**3 and miR-494 was calculated using the 2^−ΔΔCt^ approach. The primers employed are provided in Table 1.

**Table 1:** List of primers employed in RT-PCR

Name	Primer	Sequence	Size
Homo GAPDH	Forward	5′- TCAAGAAGGTGGTGAAGCAGG -3′	115 bp
	Reverse	5′- TCAAAGGTGGAGGAGTGGGT -3′	
Homo SIRT3	Forward	5’- CTTACTAGAGTGCGGCGGT-3’	220 bp
	Reverse	5’- ACAGGTCCACTCATCTTCGT-3’	
U6	Forward	5 ‘- CGCTTCGGCAGCACATATAC -3’	
	Reverse	5 ‘- AAATATGGAACGCTTCACGA -3’	
hsa-miR-494	Forward	5 ‘-TGCGCAGGTTGTCCGTGTTGTCT-3 ‘	
	Reverse	5′- CCAGTGCAGGGTCCGAGGTATT-3′	

### Western blotting (WB)

Mitochondrial, cytoplasmic and total proteins were extracted, and the corresponding kit (Beyotime) was used to detect the content. Thereafter, 25 µg of protein was subjected to sodium dodecylsulfate-polyacrylamide gel electrophoresis. The protein was transferred to a polyvinylidene fluoride film (Millipore, Billerica, Massachusetts, USA) using a semidry method. The polyvinylidene fluoride film was soaked in TBST containing 5% skimmed milk powder and sealed with a shaker for 2 h at room temperature. The blots were incubated overnight, with the primary antibodies diluted from 1:500 to 1:1000. The antibodies were used against the proteins listed below: Parkin (ab77924), PINK1 (ab23707), Bax (ab32503), Bcl-2 (ab32124), Cyt-c (ab110325), Collagen II (ab34712), Adamts5(ab41037) (Abcam, Cambridge, UK); P62 (#5114), LC3 (#2775), SIRT3 (#2627S), GAPDH (#5174), Caspase-3 (#9662) and Cleaved-caspase-3 (#9664) (Cell Signaling Technology; Danvers, Massachusetts, USA); VDAC1 (sc-32063) (Santa Cruz Biotechnology; Dallas, Texas, USA); Aggrecan (13880-1-AP) and MMP3 (17873-1-AP) (Wuhan Sanying, Wuhan, China). After rinsing the film, the proper secondary antibody was incubated through the blot for 1 h at 25 °C. The film’s gray values were analyzed after darkroom exposure using Image J software v1.46 (NIH, Bethesda, MD, USA).

#### TUNEL assay

NPCs in all groups were collected and fixed for 1 h with 4% paraformaldehyde. They were cultured with 0.1% Triton X-100 (TX) for 10 min and rinsed with PBS three times. According to the manufacturer’s instructions, the cells were stained utilizing the In Situ Cell Death Detection Kit (12156792910; Roche Applied Science, Indianapolis, IN, USA) and 40, 6-diamino-2-phenylindole (DAPI). The liquid on the slide was wiped dry using absorbent paper, and the slide was mounted with a mounting solution containing an anti-fluorescence quencher. The image was collected by observation under a fluorescence microscope (Olympus).

### Mitophagy detection assay

The mitophagy detection kit was used to detect mitophagy in NPCs. The NPCs were seeded in the logarithmic growth period on a 6-well plate covered with cell slides at 2 × 10^5^ per well. The NPCs were cultured at 37 °C overnight under 5% CO_2_ saturated humidity and treated as depicted above. Next, the cells were incubated with 100 nM Mtphagy Dye working solution for 30 min at 37 °C for mitochondrial probe staining. After that, the cells were washed twice with PBS and subjected to IL-1β for 24 h. After culturing for 24 h, 1 μmol/L LYSO DYE working solution was added to the cells and then incubated for 30 min at 37 °C. Finally, the NPCs were observed under a Laser Scanning Microscope (LSM) (ZEISSLSM780, Germany).

### Measurement of mitochondrial ROS and mitochondrial membrane potential (MMP)

Mitochondrial ROS and MMP were measured by Mito-SOX (40778ES50; Yeasen biotech, Shanghai, China) and Mitotracker (C1048, Beyotime). The NPCs were seeded in the logarithmic growth period on a 6-well plate, cultured at 37 °C overnight under 5% CO2 saturated humidity and treated as the experimental design. The NPCs were stained and fixed according to the manufacturer’s instructions. Finally, the Mito-Sox and the Mitotracker intensity were observed using the LSM.

### In situ hybridization

We used the high hybridization efficiency and specificity of oligonucleotide probes for examining miR-494 expression in NPCs. Cy3 labeled oligonucleotide probes and complementary to mature miR-494 and disruption probes were obtained from Sangon Biotech Co., Shanghai, China. ISH reactions were performed as previously described [30].

### Statistical analysis

IBM SPSS v25.0 (IBM, Armonk, New York) was used for all data analyses. Outcomes were exhibited as the mean ± SD. Student’s t test and ANOVA were for statistical analyses. Tukey’s test was used to test between group variations. Statistical significance was considered at *p* < 0.05.

## Results

### Effects of DHJSD on vitality, proliferation and extracellular matrix metabolism of NPCs exposed to IL-1β

**Fig. 1:**
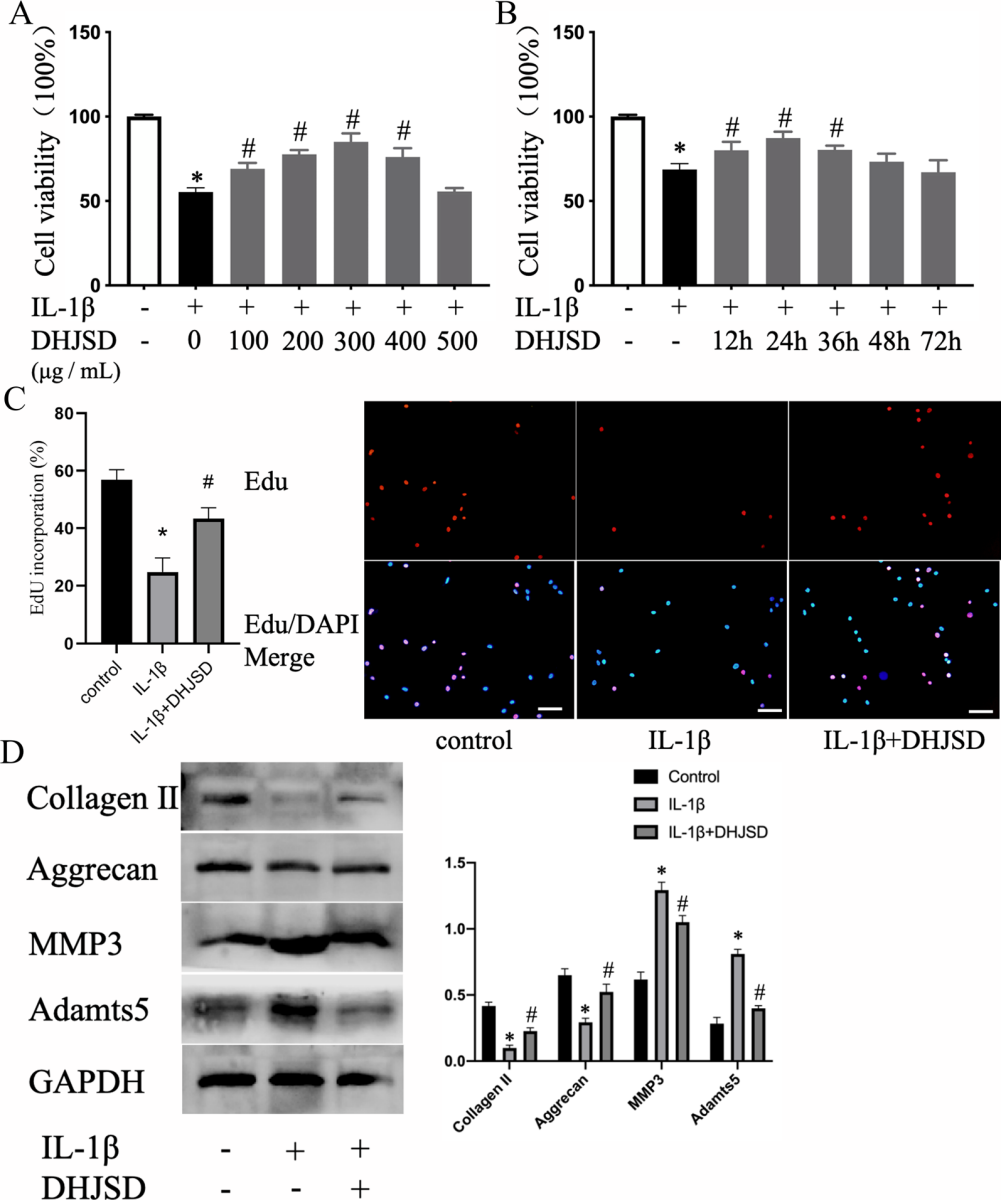
DHJSD enhances the viability of IL-1β-exposed NP cells (NPCs). **a** NPCs treated with or without IL-1β and various DHJSD concentrations for 24 h. **b** NPCs treated with or without IL-1β and 300 µg/mL DHJSD for different phases. NPCs were untreated, treated with IL-1β (10 ng/mL) alone, or with IL-1β (10 ng/mL) and DHJSD (300 µg/mL). **c** Representative fluorescence images with EDU staining and quantitative analyses of EDU-positive cells (scale bar: 50 μm, original magnification ×200) **d** The protein content of collagen II, aggrecan, MMP3 and adamts5 of NPCs treated above. All experiments were performed three times in duplicate, and data are shown as average ± SD (*n* = 3). *P < 0.05 compared with the control, #*P* < 0.05 in comparison to the IL-1β group

We examined how DHJSD affected the viability of NPCs exposed to IL-1β at various concentrations of DHJSD pretreated NPCs before incubating them for 24 h with IL-1β. DHJSD significantly enhanced the activity of NPCs treated with IL-1β in a concentration-dependent manner, and the strongest impact was observed at 300 μg/mL (Fig. 1a). Subsequently, DHJSD (300 μg/mL) was added to the NPCs pretreated with IL-1β, and the viability of NPCs was tested at various time points. The results showed that NPC viability gradually increased as the treatment time extended. After 24 h of treatment, the vitality of NPCs reached the highest point, and then, the effect of DHJSD gradually decreased (Fig. 1b). Therefore, DHJSD enhanced the viability of NPCs exposed to IL-1β in a time-concentration-dependent method. Next, NPCs were treated with 300 μg/mL DHJSD for 24 h in the follow-up experiment. IDD is characterized by a decrease in the number of NP cells and their extracellular matrix products. EDU results showed that the proliferation of the NPCs decreased after IL-1β treatment, however, the intervention of DHJSD could promote the proliferation of NPCs (Fig. 1c). Through WB, we observed that DHJSD increased the expression of collagen II and aggrecan and decreased the expression of MMP3 and adamts5, which indicates that DHJSD can reverse the decrease of extracellular matrix of NP caused by IL-1β (Fig. 1d). The above results show that DHJSD has a potentially important role in the process of IVD degeneration.

### Effects of DHJSD on IL-1β-induced apoptosis and mitochondrial dysfunction in NPCs

**Fig. 2:**
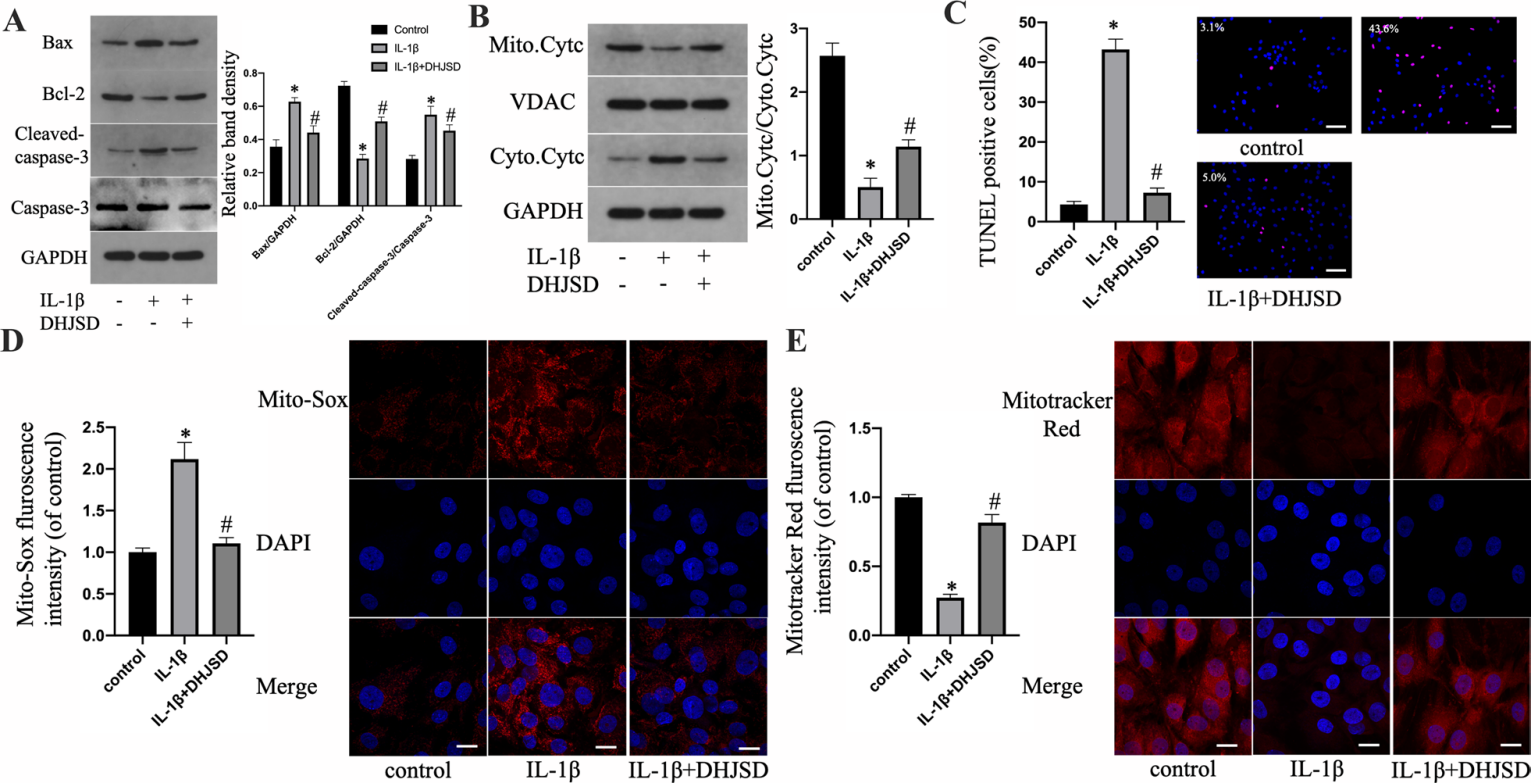
Effects of DHJSD on IL-1β-induced apoptosis and mitochondrial dysfunction in NPCs. NPCs were untreated, treated with IL-1β (10 ng/mL) alone, or with IL-1β (10 ng/mL) and DHJSD (300 µg/mL). **a** The protein content of Bcl-2 and Bax and the ratio of cleaved caspase-3/caspase-3 of NPCs treated above. **b** Representative western blotting (WB) assay and quantitation of the cytochrome c (Cyt-c) content in cytoplasmic and mitochondrial extracts. **c** Representative fluorescence images with TUNEL staining and quantitative analyses of TUNEL-positive cells (scale bar: 50 μm, original magnification ×200). **d** Representative fluorescence images with Mito-Sox and quantitative analyses of the fluorescence intensity (original magnification ×1000, scale bar: 10 μm). **e** Representative fluorescence images with Mitotracker Red and quantitative analyses of the fluorescence intensity (original magnification ×1000, scale bar: 10 μm). All experiments were performed three times in duplicate, and data are shown as average ± SD (*n* = 3). *P < 0.05 vs. the control. #*P* < 0.05 vs. the IL-1β group

The mitochondrial path modulates the cell apoptotic events through mitochondrial membrane permeabilization and subsequent proapoptotic protein release. First, the expression of apoptosis-associated proteins was detected with WB. In comparison to the control, IL-1β treatment upregulated Bax expression and the ratio of cleaved caspase-3/caspase-3 but downregulated those of Bcl-2 in human NPCs. Nonetheless, DHJSD treatment delayed the protein expression alterations in apoptotic marker genes in human NPCs induced by IL-1β exposure (Fig. 2a). We examined the cellular localization of the proapoptotic protein (Cyt-c). Their release from the mitochondria to the cytoplasm is crucial to the caspase activation apoptotic event. WB analyses proved that the ratio of mitochondrial to cytoplasmic Cyt-c was reduced by IL-1β treatment. However, DHJSD treatment delayed the trend of decreasing ratio (Fig. 2b). Second, TUNEL staining results also showed that the increased rate of apoptosis in human NPCs subjected to IL-1β alone was noticeably lessened in the DHJSD-treated group (Fig. 2c). Moreover, Mito-SOX fluorescence intensity was higher in the IL-1β-treated group than in the control group. After pretreatment with DHJSD, NPC fluorescence intensity decreased remarkably compared to that of the IL-1β-treated group (Fig. 2d). Finally, Mitotracker assay outcomes suggested that DHJSD inhibited IL-1β-induced mitochondrial membrane potential loss as well (Fig. 2e). These results demonstrated that DHJSD attenuates IL-1β-induced apoptosis and mitochondrial dysfunction in NPCs.

### DHJSD lessened IL-1β-induced NPC apoptosis by promoting mitophagy

**Fig. 3:**
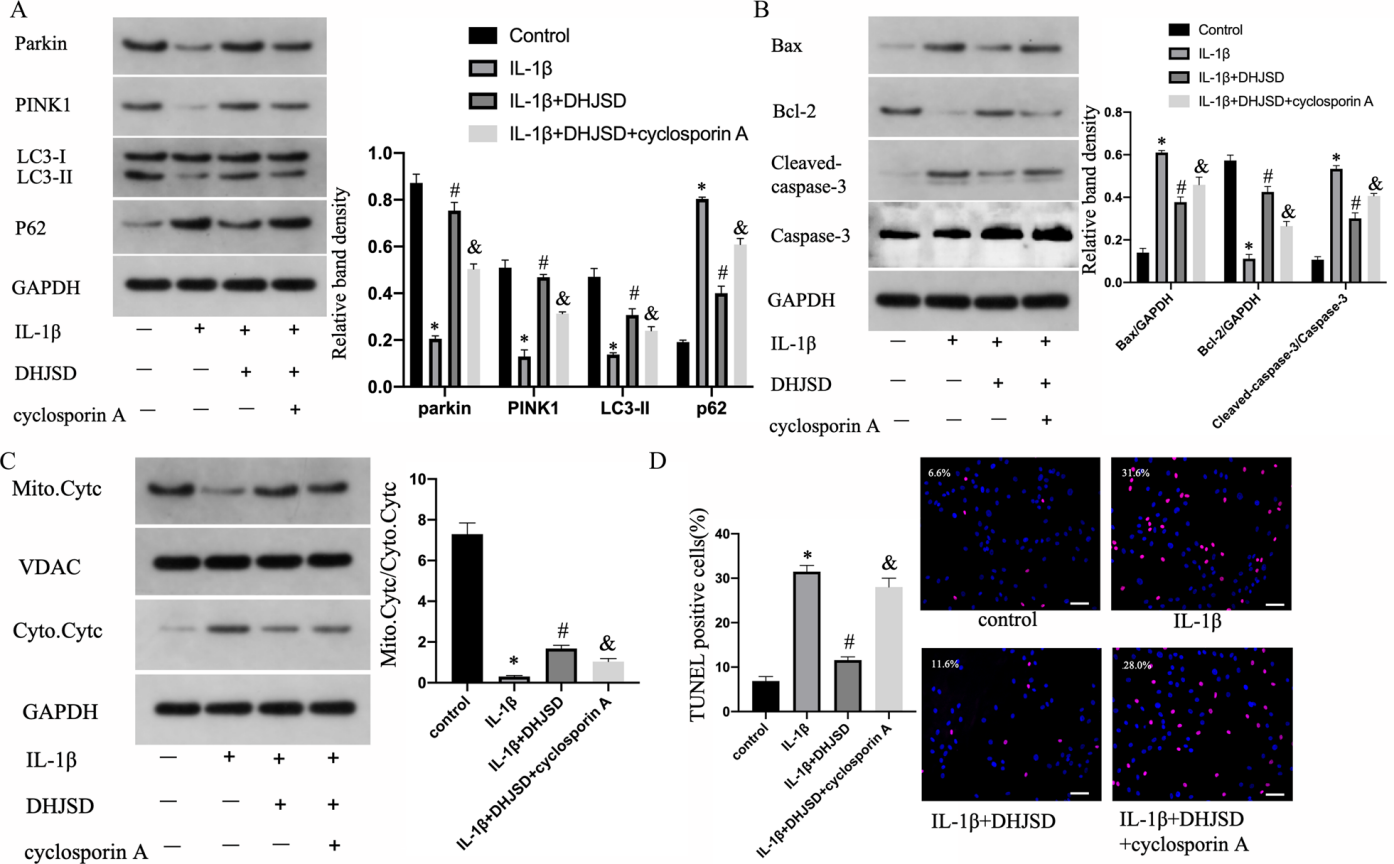
DHJSD attenuates the IL-1β-induced apoptosis of NPCs by promoting mitophagy. NP cells were untreated, treated with IL-1β (10 ng/mL) alone, with IL-1β (10 ng/mL) and DHJSD (300 µg/mL), or with cyclosporin A (1 μM), IL-1β (10 ng/mL) and DHJSD (300 µg/mL). **a** The protein content of Parkin, PINK1, LC3-II and P62 of NPCs treated above. **b** The protein content of Bcl-2 and Bax and the ratio of cleaved caspase-3/caspase-3 of NPCs treated above. **c** Representative WB assay and quantitation of the Cyt-c content in cytoplasmic and mitochondrial extracts. **d** Representative fluorescence images with TUNEL staining and quantitative analyses of TUNEL-positive cells (scale bar: 50 μm, original magnification ×200). All experiments were performed three times in duplicate, and data are shown as average ± SD (*n* = 3). *P < 0.05 vs. the control. #*P* < 0.05 vs. the IL-1β group. & < 0.05 vs. the DHJSD + IL-1β group

To evaluate how DHJSD affected mitophagy in human NPCs exposed to IL-1β, the expression of mitophagy-associated factors were identified through WB assay. IL-1β lessened the expression of Parkin, PINK1 and LC3-II but increased that of p62 (Fig. 3a). In contrast, DHJSD promoted the expression of Parkin, PINK1 and LC3-II and decreased that of p62, while those reversed distinctly when co-treated with a mitochondrial autophagy inhibitor (cyclosporin A) [18]. Next, mitochondria-dependent apoptosis was explored through WB and TUNEL assay. Cyclosporin A increased the translocation of Cyt-c limited by DHJSD treatment. Similarly, DHJSD reduced Bax expression and the ratio of cleaved caspase-3/caspase-3 but raised Bcl-2 expression; however, the impact was reversed by cyclosporin A (Fig. 3b and c). WB apoptosis results were further confirmed by TUNEL assay (Fig. 3d). These results suggest that DHJSD prevents NPCs from mitochondria-dependent apoptosis by promoting mitophagy.

### Effects of miR-494 on IL-1β-induced apoptosis and mitochondrial dysfunction in NPCs

**Fig. 4:**
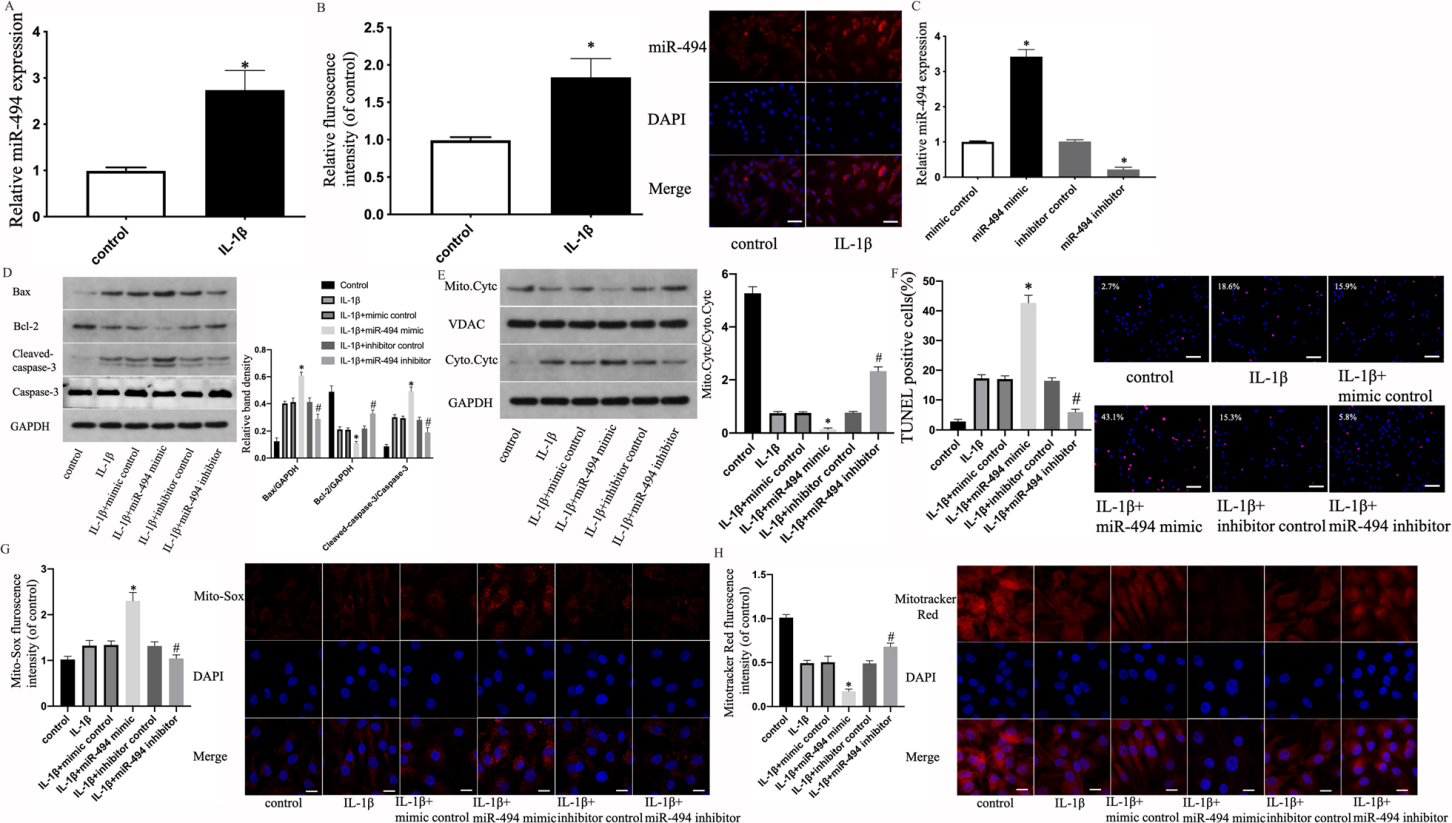
Effects of miR-494 on IL-1β-induced apoptosis and mitochondrial dysfunction in NPCs. NP cells were untreated or treated with IL-1β (10 ng/mL). **a** mRNA expression of miR-494 of NPCs treated above. **b** Representative fluorescence images with ISH and quantitative analysis (original magnification ×400, scale bar: 25 μm). **c** Transfection efficiency of miR-494 mimic and miR-494 inhibitor were measured using qRT-PCR. Data are shown as average ± SD. *P < 0.05 vs. the control. NPCs were untreated, treated with IL-1β (10 ng/mL) alone or with IL-1β (10 ng/mL) and mimic control or miR-494 suppressor, inhibitor control or miR-494 mimic. **d** The protein content of Bcl-2 and Bax and the ratio of cleaved caspase-3/caspase-3 of NPCs treated above. **e** Representative WB assay and quantitation of the Cyt-c content in cytoplasmic and mitochondrial extracts. **f** Representative fluorescence images with TUNEL staining and quantitative analyses of TUNEL-positive cells (scale bar: 50 μm, original magnification ×200). **g** Representative fluorescence images with Mito-Sox and quantitative analyses of the fluorescence intensity (original magnification × 1000, scale bar: 10 μm). **h** Representative fluorescence images with Mitotracker Red and quantitative analyses of the fluorescence intensity (original magnification ×1000, scale bar: 10 μm). All experiments were performed three times in duplicate, and data are shown as average ± SD (*n* = 3). *P < 0.05 vs. the IL-1β + mimic control. #*P* < 0.05 vs. the IL-1β + inhibitor control

Although we have previously proved that mir-494 plays an important role in IVD degeneration, the role of mir-494 in IL-1β-induced degeneration of NPCs still needs further study. MiR-494 expression in NPCs was measured using qRT-PCR. The miR-494 expression was higher in NPCs exposed to IL-1β than in the controls (Fig. 4a). The results were identified through in situ hybridization (Fig. 4b). To evaluate how miR-494 affected IL-1β-induced apoptosis and mitochondrial dysfunction in human NPCs, transfected human NPCs were exposed to IL-1β using miR-494 suppressor, suppressor control, miR-494 mimic or mimic control. Transfection efficiency of miR-494 mimic and miR-494 inhibitor was verified through qRT-PCR (Fig. 4c). First, we detected the expression of apoptosis-associated proteins using WB. Compared to the control, miR-494 overexpression upregulated Bax expression and the ratio of cleaved caspase-3/caspase-3 but downregulated Bcl-2 expression in human NPCs. However, the miR-494 inhibitor caused an inhibition of the protein expression alterations in apoptotic marker genes in human NPCs induced by IL-1β exposure (Fig. 4d). Cyt-c cellular localization was examined by WB. The results proved that the ratio of mitochondrial to cytoplasmic Cyt-c was reduced by miR-494 overexpression. However, the miR-494 inhibitor delayed the trend of decreasing ratio (Fig. 4e). Second, TUNEL staining results also showed that miR-494 overexpression raised NPC apoptosis and that low miR-494 expression delayed the IL-1β-induced apoptosis of NPCs (Fig. 4f). Additionally, Mito-SOX fluorescence intensity was higher in the group transfected with miR-494 mimic than in the control group. After pretreatment with miR-494 inhibitor, the fluorescence intensity of NPCs decreased noticeably in comparison to that of the control group (Fig. 4g). Finally, the Mitotracker assay results indicated that miR-494 regulated IL-1β-induced mitochondrial membrane potential loss (Fig. 4h).

### SIRT3 is a direct target of miR-494

**Fig. 5:**
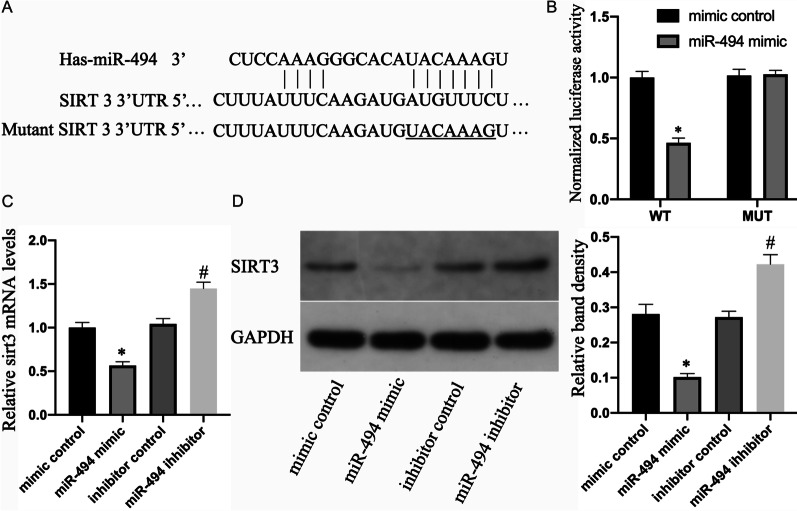
SIRT3 is a direct target of miR-494. **a** Assumed miR-494 target site in the 3′-UTR of the human SIRT3 transcript projected through bioinformatics analyses. **b** Luciferase activity in HEK 293 cells co-transfected with miR-494 mimic or mimic control and WT or MUT SIRT3 3′-UTR constructs. **c** mRNA expression of SIRT3 of NPCs treated above. **d** The SIRT3 protein content of NPCs treated above. All experiments were performed three times in duplicate, and data are shown as average ± SD (*n* = 3). *P < 0.05 vs. mimic control. #*P* < 0.05 vs. the suppressor control

The 3'-UTR of SIRT3 had a complementary sequence to the miR-494 seed sequence (Fig. 5a). Subsequently, we examined if miR-494 bound to the projected site directly in the 3'-UTR of SIRT3. The LR vector containing WT or mutated (MUT) SIRT3 3'-UTR sequence was transfected in HEK 293 cells. miR-494 overexpression effectively lessened WT luciferase activity but did not reduce the activity of the mutant reporter gene, implying that miR-494 targets **SIRT**3 3'-UTR directly (Fig. 5b). This inhibitory impact was identified through **SIRT**3 expression analyses. The results showed that miR-494 overexpression suppressed the expression of sirt3 mRNA and protein in NPCs, and miR-494 inhibition raised the **SIRT**3 mRNA and protein levels (Fig. 5c and d).

### Downregulation of miR-494 expression delayed IL-1β-induced apoptosis of NPCs by promoting SIRT3-regulated mitophagy

**Fig. 6:**
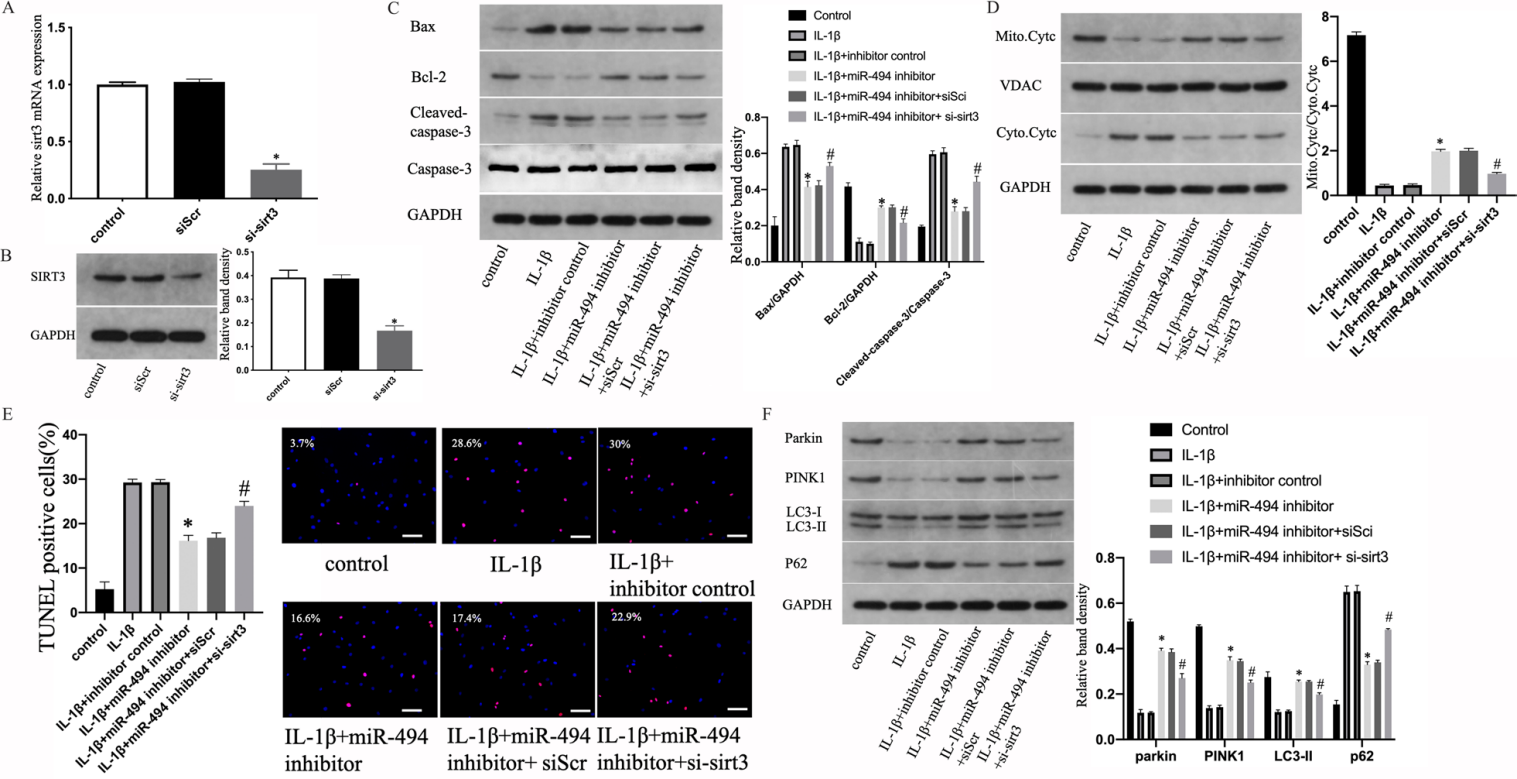
Downregulation of miR-494 expression delay IL-1β-induced apoptosis of NPCs by promoting SIRT3-regulated mitophagy. **a**, **b** Transfection efficiency of si-sirt3 was measured using qRT-PCR and WB separately. NPCs were untreated, treated with IL-1β (10 ng/mL) alone, with IL-1β (10 ng/mL) and suppressor control or miR-494 suppressor, or with IL-1β (10 ng/mL) and miR-494 inhibitor and siScr or si-sirt3. **c** The protein content of Bcl-2 and Bax and the ratio of cleaved caspase-3/caspase-3 of NPCs treated above. **d** Representative WB assay and quantitation of Cyt-c content in cytoplasmic and mitochondrial extracts. **e** Representative fluorescence images with TUNEL staining and quantitative analyses of TUNEL-positive cells (scale bar: 50 μm, original magnification ×200). **f** The protein content of Parkin, PINK1, LC3-II and p62 of NPCs treated above. All experiments were performed three times in duplicate, and data are shown as average ± SD (*n* = 3). *P < 0.05 vs. the IL-1β + suppressor control. #*P* < 0.05 vs. the IL-1β + miR-494 suppressor + siScr group

To verify whether the downregulation of miR-494 expression delayed IL-1β-induced apoptosis of NPCs by promoting **SIRT**3-regulated mitophagy, we knocked down **SIRT**3 in human NPCs by transfecting short interfering RNA against **SIRT**3 or negative control. Si-sirt3 knockout efficiency was verified through qRT-PCR and WB (Fig. 6a and b). Si-sirt3 increased the translocation of Cyt-c limited by miR-494 inhibitor. Similarly, miR-494 inhibition decreased Bax levels, the ratio of cleaved caspase-3/caspase-3 and raised Bcl-2 expression. The impacts were attenuated by the addition of si-sirt3 (Fig. 6c and d). WB results of apoptosis were further confirmed by the TUNEL assay (Fig. 6e). WB was conducted for the detection of the expression of mitophagy-related factors. As shown in Fig. 6f, we observed that the downregulation of miR-494 expression might raise the expression of Parkin, PINK1, LC3-II and decrease p62 expression, while those reversed distinctly when transfected with si-sirt3. These results suggest that **SIRT**3-regulated mitophagy is crucial to miR-494 delayed IL-1β-induced apoptosis of NPCs.

### DHJSD attenuated the IL-1β-induced apoptosis of NPCs by miR-494/SIRT3/ mitophagy signal axis

**Fig. 7:**
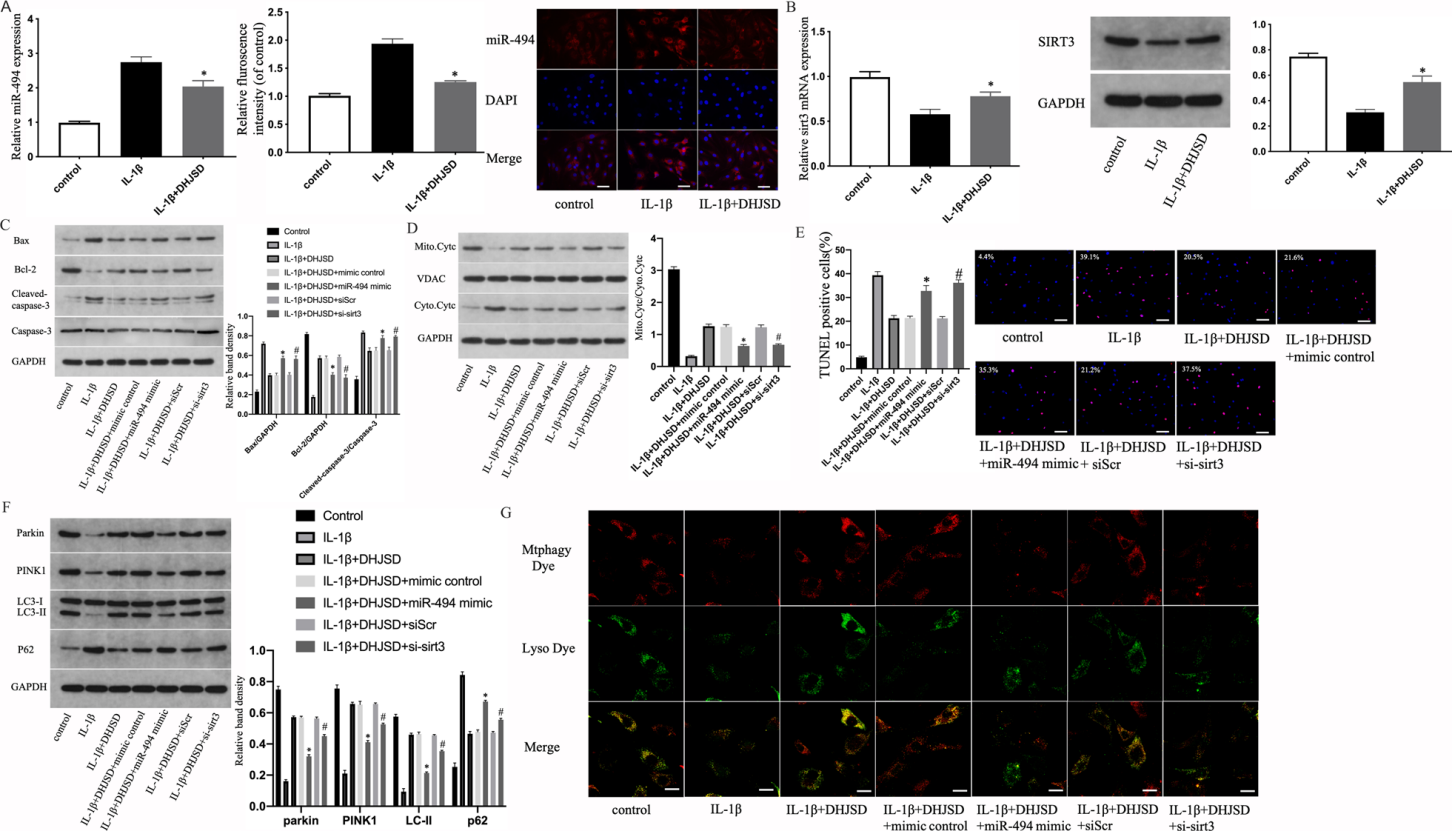
DHJSD attenuates the IL-1β-induced apoptosis of NPCs by miR-494/SIRT3/mitophagy signal axis. NP cells were untreated, treated with IL-1β (10 ng/mL) alone, or with IL-1β (10 ng/mL) and DHJSD (300 µg/mL). **a** MiR-494 expression of NP cells treated above was measured using qRT-PCR and ISH separately (scale bar: 25 μm, original magnification ×400). **b** SIRT3 expression of NPCs treated above was measured using qRT-PCR and WB separately. NPCs were untreated, treated with IL-1β (10 ng/mL) alone, with IL-1β (10 ng/mL) and DHJSD (300 µg/mL), or with IL-1β (10 ng/mL) and DHJSD (300 µg/mL) and mimic control, miR-494 mimic, siScr or si-sirt3. **c** The protein content of Bcl-2 and Bax and the ratio of cleaved caspase-3/caspase-3 of NPCs treated above. **d** Representative WB assay and quantitation of Cyt-c content in cytoplasmic and mitochondrial extracts. **e** Representative fluorescence images with TUNEL staining and quantitative analyses of TUNEL-positive cells (scale bar: 50 μm, original magnification ×200). **f** The protein content of Parkin, PINK1, LC3-II and p62 of NPCs treated above. **g** The representative images of mitophagy in NPCs treated above were detected through the mitophagy detection kit, in which red staining denotes mitophagy, green denotes lysosomes, and yellow denotes co-localization of lysosomes and mitophagy (original magnification ×1000, scale bar: 10 μm). All experiments were performed three times in duplicate, and data are shown as average ± SD (*n* = 3). *P < 0.05 vs. the IL-1β + DHJSD + mimic control. #*P* < 0.05 vs. the IL-1β + DHJSD + siScr group

Our results show that DHJSD downregulated miR-494 expression and upregulated **SIRT**3 expression than the control (Fig. 7a and b). To investigate whether DHJSD lessens IL-1β-induced apoptosis of NPCs through the miR-494/**SIRT**3/mitophagy signal axis, miR-494 mimic or si-sirt3 was transfected in NPCs. First, mitochondria-dependent apoptosis was explored through WB and TUNEL assay. DHJSD lessened Bax expression, the ratio of cleaved caspase-3/caspase-3 and raised Bcl-2 expression; however, this effect was reversed by miR-494 mimic and si-sirt3 (Fig. 7c and 7d). Likewise, we observed that miR-494 mimic and si-sirt3 increased the translocation of Cyt-c, which was limited by DHJSD treatment. WB results of apoptosis were further confirmed by the TUNEL assay (Fig. 7e). Next, we examined mitophagy in NPCs through WB assay, and the results showed that DHJSD might raise the expression of LC3-II, PINK1 and Parkin and decrease p62 expression, while those reversed distinctly when transfected with miR-494 mimic or si-sirt3 (Fig. 7f). Finally, mitophagy detection experiments showed that miR-494 mimic or si-sirt3 transfection significantly reduced mitophagy and lysosome co-localization signals compared with the DHJSD treatment group alone (Fig. 7g). These results suggest that DHJSD lessens the IL-1β-induced apoptosis of NPCs by miR-494/**SIRT**3-regulated mitophagy.

## Discussion

LBP constitutes a severe public health problem and the second most typical clinical symptom after respiratory disease [31]. The most typical reason for LBP is degenerative changes in the disk and its secondary pathological processes [32]. Nonsteroidal anti-inflammatory medicines are broadly employed in the treatment of disk degeneration. These drugs regulate effective temporary relief and inflammation of LBP; however, they cannot reverse the disk degeneration process and cause more adverse reactions [33]. Recently, TCM was observed to be highly efficacious in disk degeneration treatment and had almost no adverse reactions, sparking the interest of many researchers. In this study, we observed that DHJSD delayed the IL-1β-induced apoptosis of NPCs and mitochondrial function damage by activating mitophagy.

Studies have shown that mitochondrial apoptosis pathways are responsible for NPC apoptosis. Intracellular damage or OS conditions and excessive ROS can cause lipid peroxidation of mitochondrial membranes, Bcl-2 protein family activation to form protein holes, mitochondrial PT holes to open, apoptosis active substances released from the mitochondria to the cytoplasm, causing a downstream caspase-9 activation, and further caspase-3 activation, triggering a caspase-cascade reaction, and ultimately lead to apoptosis [10]. Ding et al. conducted a study on stressed NPC and observed that stress stimulation could damage cell mitochondria and induce apoptosis [34]. Shen studies have shown that IL-1β intervention can reduce mitochondrial membrane potential levels, reduce the Bcl-2/Bax ratio and increase Cyt-c release into the cytoplasm, which in turn causes NPCs apoptosis [35]. In this study, DHJSD reduced ROS production, stabilized the mitochondrial membrane, improved the Bcl-2/Bax ratio, reduced Cyt-c release to the cytoplasm from the mitochondria and reduced Cleaved-caspase-3 expression and thus delayed NPC apoptosis compared with the IL-1β group. Therefore, we speculate that IL-1β-induced NPC mitochondrial apoptosis is involved in the IVD degeneration course, and that DHJSD can reduce NPC apoptosis and delay IVD degeneration.

Previous studies have demonstrated a basic level of autophagy in normal NPCs. This mainly removes misfolded proteins by autophagy lysosomes to maintain cell homeostasis. After treatment with inflammatory factors such as IL-1β, the autophagy level in NPCs decreased and the apoptosis of NPCs increased. Further research shows that activating autophagy can protect NP cells from excessive apoptosis [36, 37]. Reportedly, mitophagy can degrade damaged mitochondria to maintain NP intracellular homeostasis and is responsible for the effects of OS and apoptosis. A study by Hu et al. showed that promoting Nrf2/**SIRT**3-dependent mitochondrial autophagy can suppress NPC apoptosis and improve the IVD degeneration course [21]. Lin observed that Urolithin A, a compound from natural herbs, inhibits TBHP-induced mitochondrial apoptosis in NPCs via mitophagy regulated by the AMPK signal axis [38]. Recent studies have confirmed that various TCMs exert pharmacological effects through mitophagy regulation [39, 40]. Therefore, we speculate that the anti-apoptotic impact of DHJSD is related to mitophagy. PINK1, Parkin and LC3-II are the key proteins for initiating mitochondrial autophagy, and p62 is an essential protein for autophagy degradation. We used these proteins as markers to evaluate mitophagy. We observed that DHJSD raised the expression of LC3-II, Parkin and PINK1; however, p62 expression was lessened after treatment with DHJSD. Combined with immunofluorescence results, this shows that DHJSD promotes mitophagy. Additionally, the pretreatment of mitochondrial autophagy inhibition reversed the anti-apoptotic effect of DHJSD. Based on the above results, we conclude that the anti-apoptotic effect of DHJSD is achieved through the mitochondrial pathway to promote autophagy in NPCs.

Our previous studies have shown that miR-494 is responsible for NPC apoptosis and IVD degeneration [22]. The miR-494 expression was raised in clinical samples of IVD degeneration. Decreasing miR-494 expression can reduce NPC apoptosis resulting from TNF-a. Previous in vivo experiments have shown that lowering miR-494 expression can delay the IVD degeneration process [41, 42]. Bioinformatics target projection has confirmed **SIRT**3 as an assumed target for miR-494 [43]. **SIRT**3 modulates numerous cellular courses and is a known cell-protective gene. Specifically, **SIRT**3 regulates mitophagy to prevent NPC apoptosis [19, 44]. Given the role of miR-494, **SIRT**3 and mitophagy in NPC apoptosis, we studied the specific mechanism of the miR-494/**SIRT**3/mitophagy signal axis in NPC apoptosis. We modulated miR-494 expression in NPCs and then applied the IL-1β intervention. The results showed that the differential expression of miR-494 can regulate IL-1β-induced apoptosis and mitochondrial dysfunction in NPCs. Further, we verified that **SIRT**3 was the target gene of miR-494, and the anti-apoptosis and mitochondrial autophagy effects of miR-494 were significantly weakened upon blocking **SIRT**3 expression. These results suggest that miR-494/**SIRT**3/mitophagy is responsible for the apoptosis of NP induced by IL-1β. To further explore the pharmacological effects of DHJSD, we transfected miR-494 mimic or si-sirt3 in NPCs in advance to increase miR-494 expression or reduce **SIRT**3 expression and then treated with DHJSD and IL-1β. The results showed that pretreatment of miR-494 mimic or si-sirt3 reversed the anti-apoptotic and pro-mitophagy effects of DHJSD. Based on these results, we speculate that DHJSD can delay the IL-1β-induced apoptosis of NPCs by promoting miR-494/**SIRT3-regulated** mitophagy.

This experiment had some limitations. First, although this study shows that the DHJSD plays an anti-apoptosis effect by regulating the miR-494/**SIRT**3/mitophagy signal axis, the specific mechanisms of miR-494 expression modulation by DHJSD are unknown. Our previous research showed that DHJSD could delay IVD degeneration by blocking the activity of p38MAPK [28]. Another study showed that vx745, by selectively inhibiting the activation of p38MAPK, can reduce the expression of miR-494 [43]. Therefore, we suspected that DHJSD may reduce the expression of miR-494 by inhibiting the activation of p38MAPK. However, DHJSD is a multi-herb formula containing plentiful bioactive ingredients, and its pharmacological efficacy could be derived from the synergistic actions of the multi-ingredients modulating multi-pathways in a whole system level. As for the detailed mechanism of miR-494 expression modulation by DHJSD, we need further exploration in the follow-up experiment. Second, this study was limited to the cell level, and no in vivo studies were conducted. For follow-up experiments, the rat IVD degeneration model can be used to study how DHJSD delays IVD degeneration and its mechanism of action.

## Conclusions

This study has confirmed the therapeutic impact of DHJSD on IVD degeneration. It has been proven for the first time that DHJSD plays an anti-apoptotic role by promoting mitophagy through the miR-494/**SIRT**3 signal axis and differentially regulates the expression of miR-494, which can delay IL-1β-induced apoptosis in the NPC mitochondrial pathway. These findings may provide a better understanding of the role of the miR-494/**SIRT**3/mitophagy signal axis in IVD degeneration pathogenesis and offer a theoretical foundation for TCM clinical application in treating IVD degeneration.

## Data Availability

The data supporting the study results can be obtained from the corresponding author upon reasonable request.

## Abbreviations

LBP: Low back pain
IVD: Intervertebral disk
DHJSD: Duhuo Jisheng Decoction
NP: Nucleus pulposus
NPC: Nucleus pulposus cell
Cyt-c: Cytochrome C
OS: Oxidative stress
TCM: Traditional Chinese medicine
FBS: Fetal bovine serum
CCK-8: Cell Counting Kit-8
RT-qPCR: Real-time quantitative PCR
WB: Western blotting
TUNEL: Terminal deoxynucleotidyl transferase dUTP nick end labeling
ISH: In situ hybridization
ANOVA: One-way analysis of variance
ROS: Reactive oxygen species
MMP: Mitochondrial membrane potential
